# Recommended tool compounds and drugs for blocking P2X and P2Y receptors

**DOI:** 10.1007/s11302-021-09813-7

**Published:** 2021-09-02

**Authors:** Christa E. Müller, Vigneshwaran Namasivayam

**Affiliations:** PharmaCenter Bonn, Pharmaceutical Institute, Pharmaceutical & Medicinal Chemistry, University of Bonn, An der Immenburg 4, 53121 Bonn, Germany

**Keywords:** Agonist, Antagonist, Drug, Gefapixant, P2Y receptor, P2X receptor

## Abstract

This review article presents a collection of tool compounds that selectively block and are recommended for studying P2Y and P2X receptor subtypes, investigating their roles in physiology and validating them as future drug targets. Moreover, drug candidates and approved drugs for P2 receptors will be discussed.

## Introduction

Membrane receptors activated by extracellular purines are subdivided into different subfamilies, (i) nucleotide-activated P2Y and P2X receptors [1], (ii) adenosine receptors [2, 3], and (iii) adenine-activated receptors [4], which are still poorly explored. Except for the ATP-activated P2X ion channel receptor family, all of the other purine receptors belong to the class of rhodopsin-like G protein-coupled receptors (GPCRs). There is a metabolic link between the agonists for the different receptor families, at least those activated by purines, since their physiological ligands are structurally related and can be interconverted by enzymes. For example, the nucleotide ATP, the main P2X receptor agonist, is hydrolyzed by ectonucleotidases furnishing the nucleoside adenosine which activates adenosine receptors [5].

### P2X receptors

The P2X receptors are homo- or hetero-trimeric ATP-gated ion channels [6]. Seven different subunits exist, P2X1-P2X7. Besides the homomers, heteromeric receptors exist, the best investigated one being the P2X2/3 receptor.

### P2X receptor agonists

**Fig. 1:**
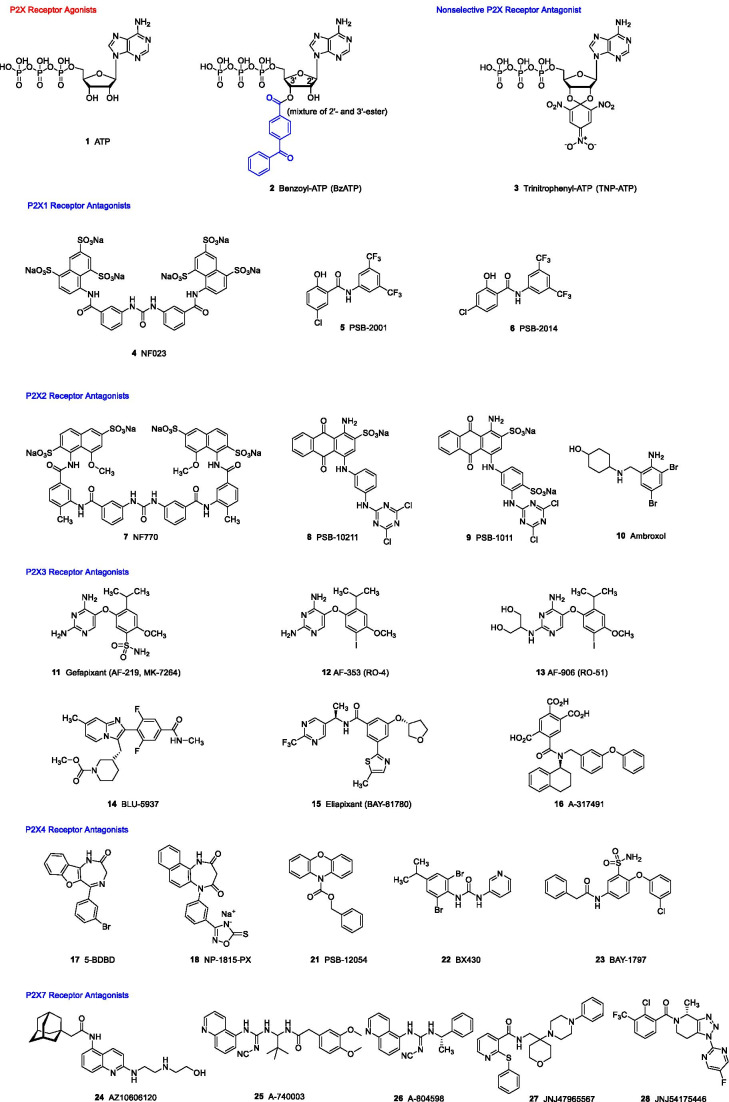
Structures of the P2X receptor agonist ATP and selected P2X receptor antagonists recommended as pharmacological tools or developed as drugs

All P2X receptors are activated by ATP (**1**, see Fig. 1), although with different potencies (see Table 1). The P2X7 receptor is the least sensitive subtype requiring high concentrations for activation, sometimes up to the millimolar range, while the P2X1 receptor is the most sensitive receptor activated by submicromolar concentrations of ATP. The P2X2, P2X3, and P2X4 receptors are activated by low micromolar concentrations. Whereas the P2X1 and P2X3 receptors show fast desensitization, the others are less quickly desensitized, and prolonged activation of the P2X7 receptor even leads to pore formation in the cell membrane. BzATP (**2**) is frequently used instead of ATP as a more potent P2X receptor agonist. This compound is, however, virtually inactive at the rat P2X4 receptor (see Table 1). Subtype-selective agonists are currently not available.

**Table 1:** Potencies of recommended P2X receptor ligands

No	Compound	EC_50_/IC_50_ values (µM) at P2X receptor subtypes^a^ (Human (h), rat (r), or mouse (m) receptor)				
		P2X1	P2X2	P2X3	P2X4	P2X7
Agonists						
**1**	ATP	0.04–0.7 (h) (fast desensitization)	0.3–8 (h)	0.04–1 (h) (fast desensitization)	0.4–10 (h)	541 (h) 2400 (m) 130 (r)
**2**	BzATP	0.02 (h)	0.75 (h)	0.08 (h)	0.5 (h) > 100 (r) 2.9 (m)	4.7 (h) 0.370 (h) 10 (r) 100 (m)
Antagonists						
Non-selective antagonist						
**3**	TNP-ATP	0.006 (h)	2 (h)	0.001 (h)	15.2 (h) 1.46 (h) 1.28 (r) 4.22 (m)	> 30 (h)
P2X1-selective antagonists						
**4**	NF023	0.21 (h) 0.24 (r)	> 50 (h)	28.9 (h) 8.5 (r) 1.4 (P2X2/3, r)	> 100 (h)	n.d.^a^
**5**	PSB-2001 (IMD 0354)	0.0192 (h)	> 10 (h)	> 10 (h)	0.156 (h)	0.175 (h)
**6**	PSB-2014	0.0231 (h)	> 10 (h)	> 10 (h)	0.209 (h)	0.196 (h)
P2X2-selective antagonists						
**7**	NF770	0.939 (r)	0.019 (r)	0.074 (r) 0.041 (P2X2/3, r)	> 10 (r)	> 10 (r)
**8**	PSB-10211	n.d	0.086 (r)	n.d	n.d	n.d
**9**	PSB-1011	0.420 (r)	0.079 (r)	0.494 (r) 1.04 (P2X2/3, r)	> 10 (r)	> 10 (r)
**10**	Ambroxol	> > 20 (h)	5.7 (h)	> > 20 (h)	> > 20 (h)	> > 20 (h)
P2X3-selective antagonists						
**11**	Gefapixant (AF-219)	> 10 (h)	0.100–0.250 (P2X2/3, h)	0.03 (h) 0.0094 (h)	> 10 (h)	> 10 (h)
**12**	AF-353 (RO-4)	> 10 (h)	> 10 (h)	0.0087 (h) 0.089 (r) 0.0389 (P2X2/3, h)	> 10 (h)	> 10
**13**	AF-906 (RO-51)	> 10 (h)	> 10 (h)	0.002 (r) 0.005 (P2X2/3, h)	> 10 (h)	> 10 (h)
**14**	BLU-5937	> 20 (h)	> 24 (h) (P2X2/3)	0.025 (h) 0.092 (r)	> 20 (h)	> 20 (h)
**15**	Eliapixant (BAY-181780)	n.d	n.d	0.008 (h)	n.d	n.d
**16**	A-317491	> 10 (h)	> 10 (h)	22 (h) 22 (r) 9 (hP2X2/3) 92 (rP2X2/3)	> 100 (h)	> 100 (h)
P2X4-selective antagonists						
**17**	5-BDBD	> 10 (r)	> 10 (r)	> 10 (r)	0.35–0.5 (h) 3.5 (r) 2.0 (m)	> 10 (r)
**18**	NP-1815-PX	> 30 (h)	7.3 (h)	> 30 (r)	0.29 (h), (similar value in r, m)	> 30 (h)
**19**	NC-2600	> 30 (h)	> 30 (h)	> 30 (h)	0.30 (h) 0.20 (r)	> 30 (h)
**20**	PSB-15417	10.3 (h)	> 10 (h)	4.14 (h)	0.0219 (h) 0.0370 (r) 0.0865 (m)	2.13 (h)
**21**	PSB-12054	6.5 (h)	> 10 (h)	> 10 (h)	0.19 (h) 2.1 (r) 1.8 (m)	> 10 (h)
**22**	BX 430	> 10 (h)	> 10 (h)	> 10 (h)	0.78 (h) 0.54 (h) > 10 (m)	> 50 (h)
**23**	BAY-1797	> 50 (h)	> 30 (h) (P2X2/3)	8.3 (h)	0.11–0.23 (h, r, m)	10.6 (h)
P2X7-selective antagonists						
**24**	AZ10606120	n.d	n.d	n.d	n.d	0.0014 (h) 0.019 (r)
**25**	A-740003	> 100 (h)	> 100 (h)	> 100 (h)	> 100 (h)	0.04–0.08 (h) 0.004–0.059 (r) 0.250 (m)
**26**	A-804598	> 100 (h)	> 100 (h)	> 100 (h)	> 100 (h)	0.010–0.060 (h, r, m)
**27**	JNJ47965567	n.d	n.d	n.d	n.d	0.005–0.0350 (h) 0.0047–0.098 (r) 0.00065 (m)
**28**	JNJ54175446	n.d	n.d	n.d	n.d	0.003 (h)

^a^for references, see text; n.d., no data reported; nevertheless, the compounds were described as being selective versus the other subtypes

### P2X receptor antagonists

**Fig. 2:**
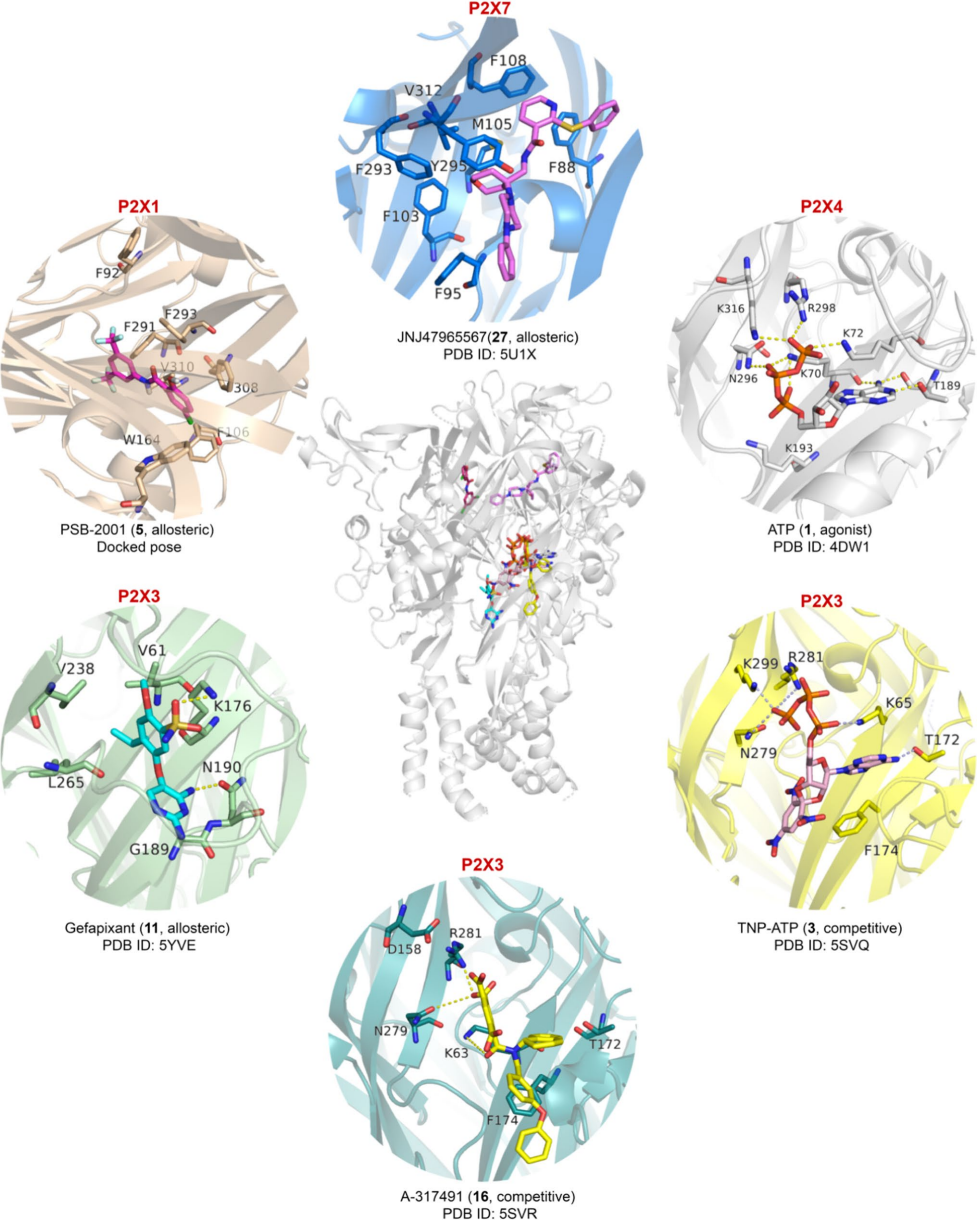
Co-crystal structures of P2X receptors and their ligands, the agonist ATP and a variety of orthosteric and allosteric antagonists. For the P2X1 receptor antagonist, a docked pose is shown. For references see text

A frequently used ATP-derived, non-selective competitive P2X receptor antagonist is TNP-ATP (**3**, see Figs. 1 and 2, and Table 1), which is particularly potent at P2X1 and P2X3 receptors, and an X-ray co-crystal structure with the human P2X3 receptor has been obtained (see below). Selective P2X receptor antagonists have been developed for P2X1, P2X2, P2X3, P2X4, and P2X7 receptors (see Table 1) [6–10].

### P2X1 receptor antagonists

The symmetrical polysulfonated naphthyl derivative NF023 (**4**, Fig. 1 and Table 1) derived from suramin appears to act as a competitive P2X1 receptor antagonist [11]. Due to its polyanionic character, it is well soluble in water, but its selectivity is limited since it also blocks the P2X3 receptor at somewhat higher concentration. The first relatively potent, selective P2X1 receptor antagonists have recently been described, salicylamide derivatives **5** and **6** [12]. These compounds act as allosteric inhibitors, and their binding site was proposed by docking studies to be located in the extracellular domain (see Fig. 2). They can also inhibit P2X4 and P2X7 receptors at higher concentrations (see Table 1).

### P2X2 receptor antagonists

Compound NF770 (**7**), another symmetrical suramin-derived polysulfonated naphthyl derivative, was described as a potent and relatively selective competitive antagonist of rat P2X2 receptors [13]. However, it also blocks the rat P2X3 receptor at somewhat higher concentration. The Reactive Blue 2 (RB2)-related sulfoanthraquinone derivatives PSB-10211 (**8**) and PSB-1011 (**9**) act as potent, but moderately selective rat P2X2 receptor antagonists, probably displaying an allosteric mechanism of inhibition [14]. Recently, ambroxol (**10**) a bronchosecretolytic drug, which is frequently used to treat bronchial diseases since more than 40 years, was discovered to block the human P2X2 receptor with low micromolar potency [15]. Based on its structure, it likely displays an allosteric mechanism of inhibition. The newly discovered P2X2 receptor blockade might contribute to ambroxol’s biological activity since plasma concentrations should be sufficiently high [16]. Ambroxol likely acts as a multi-target drug also interacting with other targets [17–19].

### P2X3 receptor antagonists

**Fig. 3:**
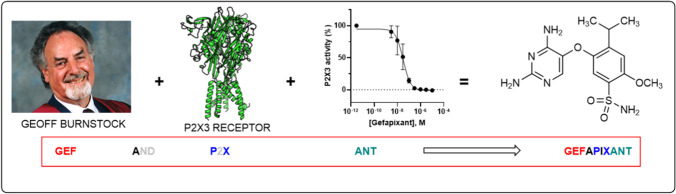
Naming of the clinically most advanced, most promising P2X receptor antagonist

The P2X3 receptor has been in the focus of drug development efforts by pharmaceutical companies since decades due to their expected analgesic and anti-inflammatory effects [19], and therefore, a number of potent P2X3 receptor antagonists have been developed (Fig. 1) [20–22]. The orally administered potent and selective allosteric P2X3 antagonist gefapixant (AF-219, **11**) successfully passed a phase III clinical trial for the treatment of refractory chronic cough [23] and is likely to become the first P2X3 receptor antagonist that will be approved as a drug [24]. This has revived the field, and more P2X3 receptor antagonists are now being developed and clinically evaluated for various indications (see, e.g., [21, 25–27]). The etymology of the international non-proprietary name (INN) “gefapixant” is explained in Fig. 3 — in fact, it contains part of Geoffrey Burnstock’s name. A co-crystal structure of gefapixant with the human P2X3 receptor has been solved [28]. Its allosteric binding site is located near the orthosteric ATP binding site (see Fig. 2). Structurally related potent P2X3 receptor antagonists include AF-353 (**12**) and AF-906 (**13**), all of which are highly potent and perorally bioavailable. The more lipophilic antagonist **12** is even brain-permeant. Antagonists **11–13** also block the heteromeric P2X2/3 receptor subtype at somewhat higher concentrations. In contrast, the imidazopyridine derivative BLU-5937 (**14**) was reported to be selective for the homomeric P2X3 receptor; it is currently evaluated in clinical trials for the treatment of chronic cough and pruritus [29]. Eliapixant (BAY-181780, **15**), another potent and selective P2X3 receptor antagonist [21, 27, 30], has completed a phase 2a clinical trial for the treatment of refractory chronic cough [25] and is further clinically evaluated for the treatment of overactive bladder; another pursued indication is neuropathic pain. A further P2X3 antagonist with undisclosed structure (S-600918) is evaluated in clinical studies [10, 31]. Besides negative allosteric modulators, orthosteric P2X3 receptor antagonists have also been described, e.g., A-317491 (**16)** [32], which bears three carboxylate functions that interact with basic amino acids in the triphosphate binding pocket as demonstrated by an X-ray co-crystal structure (see Fig. 2) [33].

### P2X4 receptor antagonists

5-BDBD (**17**, Fig. 1 and Table 1), a diazepinone derivative, is a moderately potent and selective allosteric P2X4 receptor antagonist [34]; its main drawback is its low water solubility. A structurally related diazepinedione derivative, NP-1815-PX (**18**), developed by Japanese researchers around Kazuhide Inoue, is well water-soluble as a sodium salt; but due to its polarity, it does not penetrate into the central nervous system (CNS) [35]. The first P2X4 antagonist, which was evaluated in a clinical trial (phase I), is NC-2600 (**19**, structure undisclosed) [36]; no further development has been reported despite the positive outcome of the study. A potent, brain-permeable allosteric P2X4 receptor antagonist that is potent at human, rat, and mouse P2X4 receptors and highly selective versus the other P2X receptor subtypes is PSB-15417 (**20**, structure undisclosed) developed in our group. It showed high efficacy in animal models of neuropathic pain [37]. The *N*-(benzyloxycarbonyl)phenoxazine derivative PSB-12054 (**21**) [38] and the urea derivative BX430 (**22**) [39], moderately potent allosteric P2X receptor antagonists with limited water solubility, are more potent at human than at rodent receptors and can therefore only be recommended for studying the human P2X4 receptor. In contrast, the recently described sulfonamide derivative BAY-1797 (**23**) [40] is similarly potent at human, rat, and mouse P2X4 receptors showing good P2X4 selectivity but moderate potency. The allosteric antagonist is well water-soluble. Due to its polar character, **23** does not penetrate well into the brain and is only peripherally active.

### P2X7 receptor antagonists

The development of P2X7 receptor antagonists is most advanced since the receptor has early been regarded as a promising drug target for treating inflammatory diseases, but the first clinical trials were not successful [10]. Most of the described potent and selective antagonists are negative allosteric modulators, many of which have been optimized with regard to their pharmacokinetic properties [41, 42]. Structurally diverse compounds are available, e.g., **24–28**. Antagonist AZ10606120 **24** has a particularly high water solubility (25 mM), while compounds **26**–**28** have been described to penetrate well into the brain. JNJ47965567 (**27**) and related antagonists were co-crystallized with the human P2X7 receptor and shown to interact with a peripheral binding site far from the orthosteric ATP binding region (see Fig. 2) [43]. JNJ54175446 (**28**) is a brain-permeable drug that is clinically evaluated for the treatment of major depression and bipolar disorders [44].

### Ortho- and allosteric ligand binding sites

The first crystal structure of a P2X receptor in complex with its agonist ATP (Fig. 2), namely, that of the zebrafish P2X4 receptor, confirmed the trimeric structure and the location of the orthosteric binding site within each of the three subunits [45, 46]. ATP was found to bind in an unusual U-shaped conformation (see Fig. 2). Important interactions include (i) polar interactions between the anionic γ-phosphate group of ATP with basic amino acid residues (K72, R298, and K316) and (ii) hydrogen bond interactions of the N^6^-amino group of ATP with K70 and T189 [47]. Structures of the human P2X3 receptor in complex with the orthosteric antagonists TNP-ATP (**3**) and A-317491 (**16**) provided molecular insights in the binding of competitive antagonists [33]. Interestingly, TNP-ATP, a derivative of ATP that is substituted at the ribose-2’- and 3’-hydroxy groups, displays partly similar interactions and binds in a similar position as the agonist ATP. Its additional lipophilic, aromatic trinitrophenyl substituent binds in a deep hydrophobic cleft between two receptor subunits. A very similar binding position is observed for A-317419 (**16**), in which the carboxylate functions attached to a phenyl ring adopt the role of the nucleotidic phosphate groups. The particular orientation of the lipophilic substituents, present in the antagonists **3** and **16**, and their interactions restrict the upward movement of the dorsal fin domain which is required for channel opening.

Besides the orthosteric binding site for ATP, the P2X receptors harbor additional allosteric binding sites which can result in a modulation of the agonist activity (see Fig. 2). The crystal structure of the human P2X3 receptor in complex with the negative allosteric modulator gefapixant (**11**) revealed its binding between the left flipper and the lower body of the receptor, not too far from the orthosteric site [28]. The inhibitor forms hydrogen bond interactions with the main chain of N190 and the side chain of K176 supported by hydrophobic interactions with V61, V238, and L265 (see Fig. 2). The crystal structures of the human P2X7 receptor with the antagonist JNJ47965567 (**27**) and four other antagonists identified a common allosteric drug binding site in a groove formed between two adjacent subunits in the upper body domain of the receptor [43]. The allosteric drug binding pocket becomes narrower upon ATP binding, a conformational rearrangement that is crucial for P2X7 receptor channel opening. This action is blocked by antagonists such as **27**, which binds deep within the cavity, primarily mediated by hydrophobic interactions with F88, F95, F103, M105, F293, and V312 (see Fig. 2). To provide a structural hypothesis for the recently described series of allosteric P2X1 receptor antagonists, a homology model was generated based on the crystal structures of the P2X7 receptor [12]. The putative binding mode of PSB-2001 (**5**) and its close analogs partly overlaps with the allosteric binding site identified by the crystal structures of the antagonist-bound P2X7 receptor. The aniline moiety of PSB-2001 is proposed to be embedded forming hydrophobic interactions with F92, F291, F293, F308, and V310, and the salicylate moiety is likely surrounded by another set of hydrophobic residues, F106 and W164 (see Fig. 2) [12].

### P2Y receptors

**Fig. 4:**
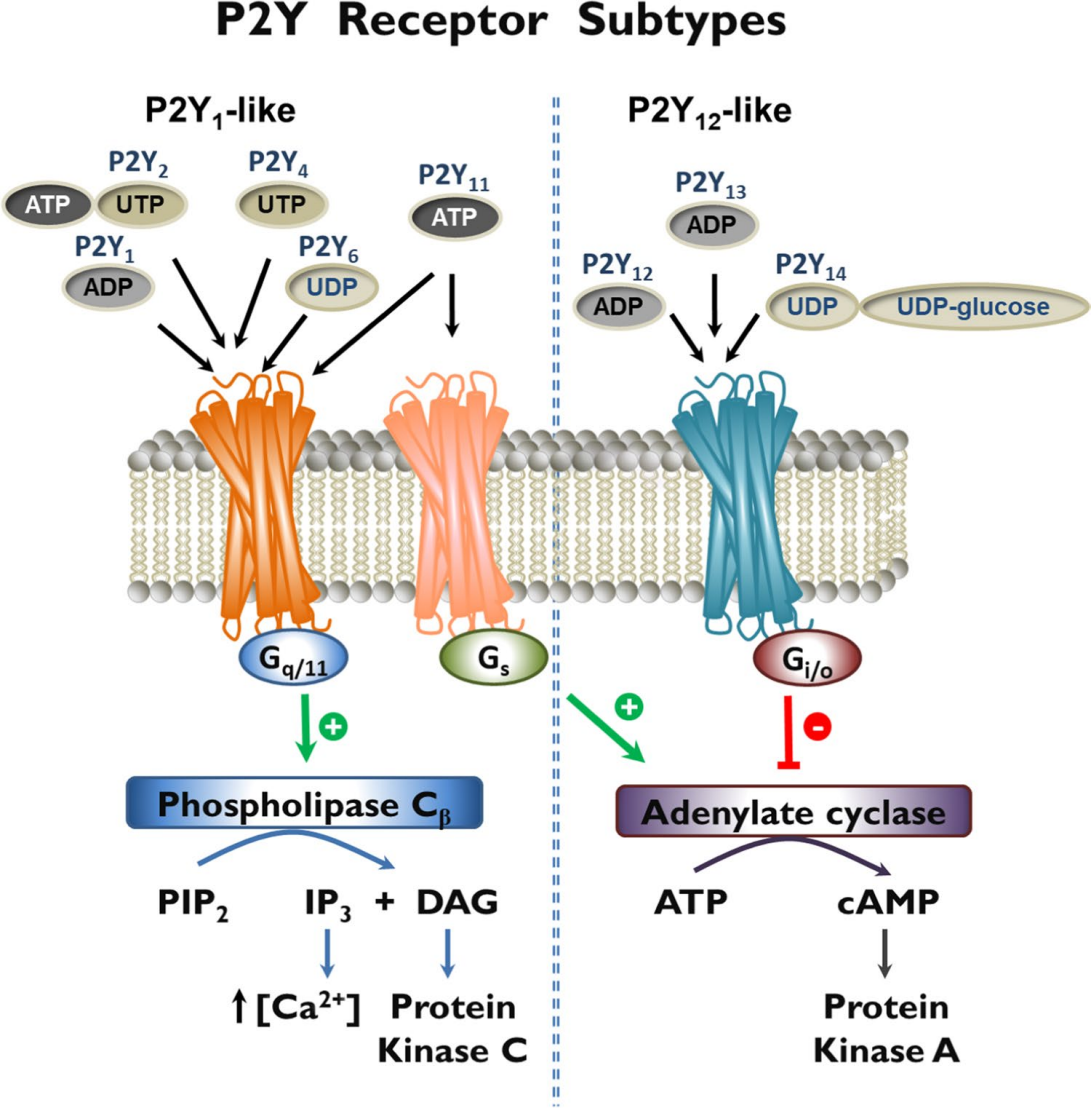
P2Y receptor subtypes

P2Y receptors are G protein-coupled receptors belonging to the δ-branch of the rhodopsin-like receptor family. They are activated by nucleotides such as ATP, ADP, UTP, UDP, or UDP-glucose depending on the receptor subtype (see Fig. 4) [48]. The P2Y_1_-like receptors include P2Y_1_ (activated by ADP), P2Y_2_ (ATP, UTP), P2Y_4_ (UTP), P2Y_6_ (UDP), and P2Y_11_ (ATP). The P2Y_12_-like receptor family consists of three subtypes, P2Y_12_ (ADP), P2Y_13_ (ADP), and P2Y_14_ (UDP, UDP-glucose). P2Y_1_, P2Y_2_, P2Y_4_, and P2Y_6_ couple to Gq proteins leading to the activation of phospholipase C, release of inositol trisphosphate (IP_3_), and intracellular calcium mobilization. The P2Y_11_ receptor is only present in primates; it can activate phospholipase C via Gq proteins, as well as adenylate cyclase via Gs proteins. P2Y_12_, P2Y_13_, and P2Y_14_ receptors inhibit adenylate cyclase via G_i_ proteins (see Fig. 4). Some of the receptors can also be activated by dinucleotides, e.g., diadenosine tetraphosphate (Ap_4_A). Comprehensive reviews on P2Y receptor agonists and antagonists have recently appeared [7, 10, 49, 50]. Many of the nucleotides and their analogs, which have been developed as selective agonists for specific subtypes, are metabolically unstable under in vivo conditions. Therefore, it is advisable to study P2Y receptors using the frequently more stable, subtype-selective P2Y receptor antagonists, as far as available.

### P2Y_1_ receptor antagonists

**Fig. 5:**
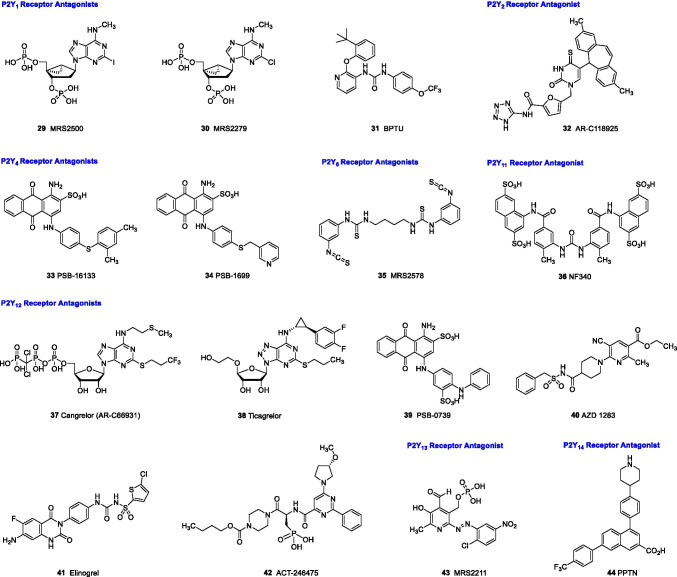
Structures of selected P2Y receptor antagonists recommended as pharmacological tools or developed as drugs

**Fig. 6:**
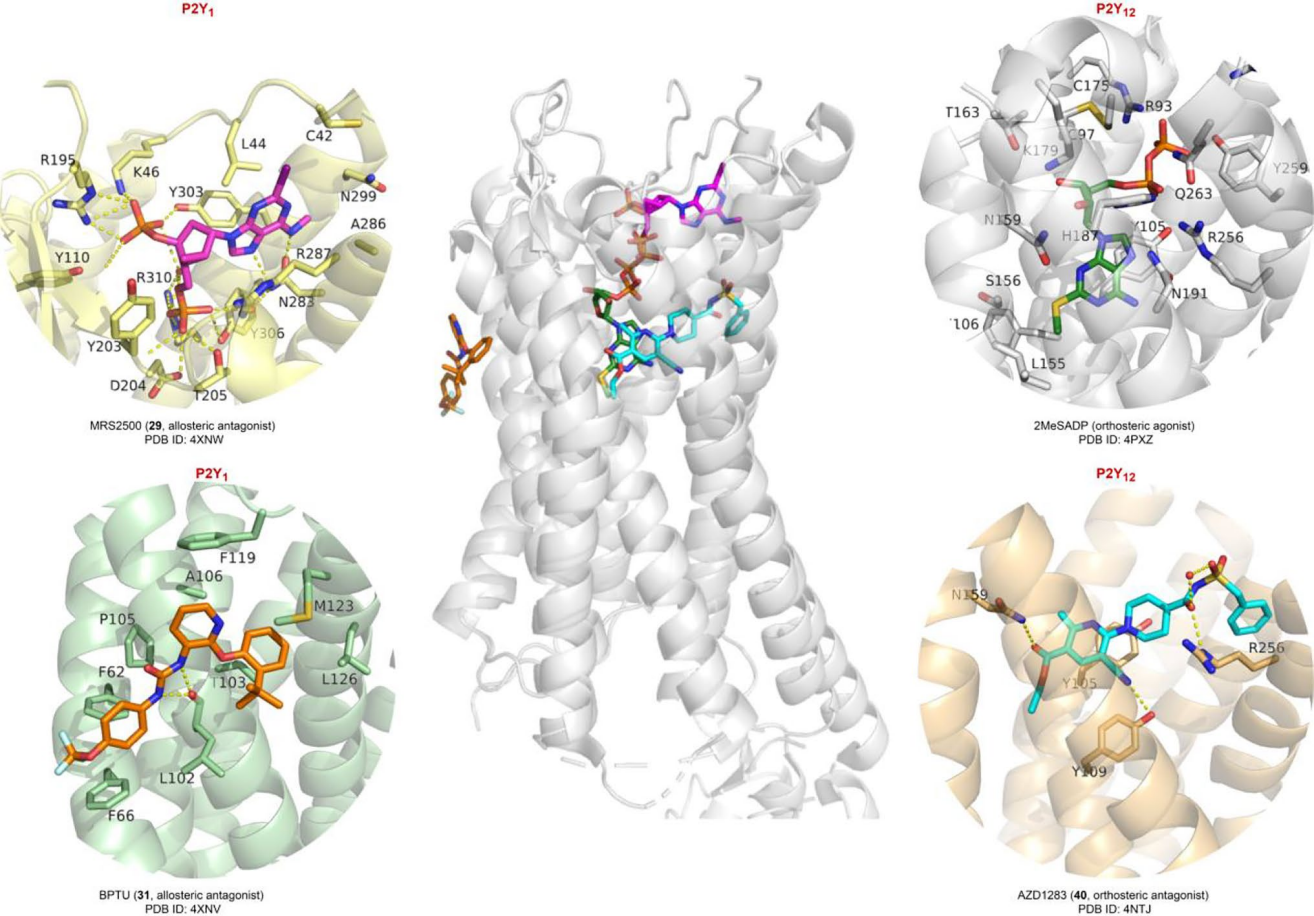
Co-crystal structures of P2Y receptors and their ligands. For references see text

The nucleotide derivatives MRS2500 (**29**) and MRS2279 (**30**) are potent and selective antagonists that bind close to the orthosteric ADP binding site but have mostly allosteric interactions as shown in a co-crystal structure of **29** with the human P2Y_1_ receptor (see Table 2, Figs. 5 and 6) [51, 52]. In comparison to the antagonist **29**, which bears only one phosphate group at the 5’-OH group and another one at the 3’-OH group of the adenosine analog (see Fig. 5), the agonist ADP and its derivative 2-methylthio-ADP (2MeSADP) are predicted to display a different binding mode, binding in a deeper orthosteric binding pocket [52]. This suggestion was based on molecular docking and molecular dynamics simulation (MD) studies taking into account an X-ray structure of the agonist-bound P2Y_12_ receptor, which is activated by the same agonists, ADP and 2MeSADP [52, 53]. The binding of the β-phosphate group of ADP to the basic amino acid residues R287, D304, and R310 is predicted to overlap with the binding of the 3’-phophate group of the antagonist MRS2500 (**29**) (see Fig. 6). The binding of an agonist is expected to contract the orthosteric binding pocket, thereby inducing an inward shift of the extracellular part of helix VI (stretching of the bent conformation) towards the center of the receptor. The antagonists **29** and **30**, bearing phosphoric acid ester functions, are metabolically not very stable and are therefore not the first choice for in vivo studies but can be excellent tools for in vitro experiments.

**Table 2:** Potencies of recommended P2Y receptor antagonists

No	Compound	K_i_ or IC_50_ values (µM)^a^ (Human (h) or rat (r) receptor)							
		P2Y_1_	P2Y_2_	P2Y_4_	P2Y_6_	P2Y_11_	P2Y_12_	P2Y_13_	P2Y_14_
P2Y_1_-selective antagonists									
**28**	MRS2500	0.00078–0.0084 (h)	n.d	n.d	n.d	n.d	n.d	n.d	n.d
**29**	MRS2279	0.0025–0.052 (h)	> 30 (h)	> 30 (h)	> 30 (h)	> 30 (h)	n.d	n.d	n.d
**30**	BPTU	0.006 (h)	> 15 (h)	n.d	> 15 (h)	> 15 (h)	> 70 (h)	n.d	3.5 (h)
P2Y_2_-selective antagonists									
**31**	AR-C118925 (= AR-C118925XX)	36.9 (h)	0.0574–0.716 (h) 0.291 (r)	37.1 (h)	30.4 (h) > 100 (r)	4.02 (h)	33.7 (h)	n.d	> 3 (h)
P2Y_4_-selective antagonist									
**32**	PSB-16133	5.48 (h)	8.54 (h)	0.233 (h)	12.5 (h)	n.d	2.41 (h)	n.d	n.d
**33**	PSB-1699	ca. 20 (h)	13.1 (h)	0.409 (h)	> 100 (h)	n.d	3.59 (h)	n.d	n.d
P2Y_6_-selective antagonist									
**34**	MRS2578	> 10 (h)	> 10 (h)	> 10 (h)	0.037 (h) 0.098 (r)	> 10 (h)	n.d	n.d	n.d
P2Y_11_-selective antagonist									
**35**	NF340	> 10 (h)	> 10 (h)	> 10	> 10 (h)	0.0724–0.372 (h)	> 10 (h)	n.d	n.d
P2Y_12_-selective antagonists									
**36**	Cangrelor (AR-C69931, AR-C69931MX)	> 3 (h)	> 3 (h)	> 3 (h)	> 3 (h)	0.0046 (h)	0.0004 (h)	> 3 (h)	> 3 (h)
**37**	Ticagrelor (AZD6140)	n.d	n.d	n.d	n.d	n.d	0.002–0.014 (h)	n.d	n.d
**38**	PSB-0739	n.d	n.d	n.d	n.d	n.d	0.0004–0.0249 (h)	n.d	n.d
**39**	AZD1283	n.d	n.d	n.d	n.d	n.d	0.011–0.025 (h)	n.d	n.d
**40**	Elinogrel	n.d	n.d	n.d	n.d	n.d	0.023 (h)	n.d	n.d
**41**	Selatogrel (ACT-246475)	> 10 (h)	> 10 (h)	> 10 (h)	> 10 (h)	> 10 (h)	0.001 (h)	> 10 (h)	n.d
P2Y_13_-selective antagonist									
**42**	MRS2211	> 10	n.d	n.d	n.d	n.d	> 10	0.501– 1.07 (h)	n.d
P2Y_14_-selective antagonists									
**43**	PPTN	> 1 (h)	> 1 (h)	> 1 (h)	> 1 (h)	> 1 (h)	> 1 (h)	> 10 (h)	0.000434 (h)

^a^for references, see text; n.d., no data reported; nevertheless, the compounds were described as being selective versus the other subtypes

The urea derivative BPTU (**31**) is an allosteric antagonist that binds at the periphery of the receptor, far from the ADP binding site and close to the phospholipid interface (see Fig. 6) [52]. While the nucleotidic antagonists are highly water-soluble, **31** is lipophilic and displays only limited water solubility.

### P2Y_2_ receptor antagonists

The most potent and selective P2Y_2_ receptor antagonist available so far is the nucleotide analog AR-C118925 (**32**) [54–58]. The competitive antagonist, whose structure imitates that of an uracil nucleotide, displays relatively high potency at human and rodent P2Y_2_ receptors and is well soluble in buffer at pH 7.4 due to its negatively charged tetrazolate ring which mimics a phosphate group [56]. Because of its high polarity, **32** has low peroral bioavailability and cannot penetrate into the brain. The compound showed high metabolic stability in human and mouse liver microsomes and is therefore suitable for in vivo studies but will have to be parenterally applied [52]. The P2Y_2_ receptor antagonist has already been used in a number of in vitro and in vivo studies to investigate the role of the receptor in health and disease (e.g., [59–63]).

### P2Y_4_ receptor antagonists

Only few antagonists are available to study the P2Y_4_ receptor. PSB-16133 (**33**) and PSB-1699 (**34**) show P2Y_4_ receptor inhibition at submicromolar concentrations combined with selectivity versus the other P2Y receptor subtypes (see Fig. 5 and Table 2) [57]. PSB-16133 is somewhat more potent, while PSB-1699 is more selective for the P2Y_4_ receptor. Both compounds are sulfonated, negatively charged anthraquinone derivatives. These polar compounds display relatively good water solubility but can probably not penetrate cell membranes.

### P2Y_6_ receptor antagonists

The isocyanato-substituted dimeric thiourea derivative MRS2578 (**35**) acts as an irreversible inhibitor of P2Y_6_ receptors [64]. The compound is not ideal due to its low water solubility and high reactivity, but nevertheless, it has already been utilized for a number of in vitro and in vivo studies (e.g., [65–69]).

### P2Y_11_ receptor antagonists

Several suramin derivatives have been described to block the P2Y_11_ receptor with relatively high potency and selectivity [70]. One of the best antagonists so far is NF340 (**36**) (see Table 2 and Fig. 5), which acts as a competitive antagonist [70]. The compound bears four sulfonate functions and is therefore highly polar, deprotonated at pH 7.4, and well water-soluble in its deprotonated form.

### P2Y_12_ receptor antagonists

The P2Y_12_ receptor is an important target for antithrombotic drugs, and P2Y_12_ antagonists are widely used to prevent cardiac infarction and stroke [71]. Therefore, a variety of P2Y_12_ receptor antagonists belonging to different chemical classes have been developed to date, and further drug discovery in this field is still ongoing.

The therapeutically used thienotetrahydropyridine derivatives clopidogrel and prasugrel are prodrugs of irreversibly acting allosteric P2Y_12_ receptor antagonists. They have to be metabolized by cytochrome P450 enzymes in the liver before they can react with a cysteine residue in the extracellular domain of the P2Y_12_ receptor that is not part of the orthosteric binding site, forming a stable disulfide bond [72, 73]. Therefore, these prodrugs are only suitable for systemic application in vivo where they can be metabolized in the liver. Only peripheral P2Y_12_ receptors will be blocked, preferably on thrombocytes.

For pharmacological studies of P2Y_12_ receptors, several direct, competitive antagonists are available, several of which are approved as therapeutic antithrombotic drugs endowed with the required pharmacokinetic properties. Cangrelor (**37**) is a therapeutically used nucleotide analog derived from ATP acting as a competitive antagonist and displaying very high potency and selectivity versus most P2Y receptor subtypes (see Fig. 5 and Table 2) [74]; however, it also potently blocks the P2Y_13_ receptor [75]. The compound is very polar and well water-soluble due to its nucleotidic structure; it can only be applied parenterally since it is not able to penetrate membranes and is therefore not orally bioavailable. Ticagrelor (**38**) is the result of cangrelor’s further optimization. It lacks the phosph(on)ate groups, is therefore less polar, and shows good peroral bioavailability. The compound is a potent competitive P2Y_12_ receptor antagonist with inverse agonistic activity that is widely employed as an antithrombotic drug. However, it was shown to additionally block the human adenosine A_3_ receptor and the equilibrative nucleoside transporter ENT-1 which might be responsible for side effects [76–80].

A different class of potent, competitive P2Y_12_ receptor antagonists bears a sulfoanthraquinone scaffold [81, 82]. PSB-0739 (**39**) was the most potent compound in this series with (sub)nanomolar potency and high selectivity. Due to its sulfonate groups, it is well water-soluble but not orally bioavailable. The compound has been applied in a number of experimental studies in vitro and in vivo to block P2Y_12_ receptors (e.g., [83–86]).

The acylsulfonamide derivative AZD 1283 (**40**) is another experimental drug that shows good water solubility [72, 87, 88]. The pK_a_ value of its sulfonamide function is relatively low (4.6), and the compound is therefore deprotonated at pH 7.4. The competitive antagonist was co-crystallized with the human P2Y_12_ receptor (see Fig. 6) [72]. AZD 1283 binds in an elongated conformation making strong polar as well as hydrophobic interactions with the amino acid residues in the orthosteric binding pocket. Another sulfonamide derivative related to **40** is elinogrel (**41**) which was reported to also display high water solubility [89].

Selatogrel (ACT-246475, **42**) belongs to a different class of competitive P2Y_12_ receptor antagonists with a peptide-like structure and a phosphonate function [89–92]. The drug itself is very polar and is therefore subcutaneously applied; an orally bioavailable prodrug, ACT-281959, has also been developed. Selatogrel is currently evaluated in clinical trials in patients with stable coronary artery disease and in patients with acute myocardial infarction.

Due to their polar nature, none of the currently available P2Y_12_ receptor antagonists penetrates well into the brain; if blocking of brain P2Y_12_ receptors is required, the drugs have to be directly injected. The development of a brain-permeant P2Y_12_ receptor antagonists would be highly desirable to study their potential for treating brain diseases, e.g., as neuroprotective drugs.

### P2Y_13_ receptor antagonists

The P2Y_13_ receptor is the least investigated P2Y receptor subtype. It is most closely related to the P2Y_12_ receptor, and the P2Y_12_ receptor antagonist cangrelor (**37**), an ATP analog, was reported to also block the P2Y_13_ receptor subtype with high potency [75].

The pyridinecarboxaldehyde derivative MRS2211 (**43**), which bears a phosphate group, was described as a moderately potent competitive P2Y_13_ receptor antagonist, but its selectivity is unclear [93]. Since it bears an aldehyde function and a phosphoric acid ester group, its stability can be expected to be limited, and the compound is recommended for in vitro studies only.

### P2Y_14_ receptor antagonists

Potent and selective competitive P2Y_14_ receptor antagonists have been developed [94, 95]. The commercially available antagonist PPTN (**44**), a naphthalenecarboxylic acid derivative, shows very high potency and selectivity [96].

## Conclusions

The field of purinergic receptors, which was widely opened, nurtured, and continuously promoted by Geoffrey Burnstock, has now attracted researchers from many different areas of research. One of the latest disciplines that discovered the importance of purinergic signaling was immunology. And again, Burnstock saw its relevance long ago and paved the way [97]. It is now generally recognized that ATP and purinergic signaling are important for all processes in the body, in health, and especially in disease, where ATP can be described as a danger signal. P2Y_12_ receptor antagonists have become essential antithrombotic drugs. The first P2X3 receptor antagonist, gefapixant, named after Geoff Burnstock, is expected to be soon approved for the treatment of chronic cough. Other drugs interacting with P2 receptors are currently evaluated in clinical trials, and many more will be developed. The availability of suitable tool compounds and drugs will help to advance basic research and target validation in this field. While a number of tool compounds for studying P2X and P2Y receptors are available, they often lack drug-likeness, e.g., due to the presence of several negative charges associated with high polarity, large molecular weights, or moderate selectivity. Despite enormous progress in recent years, there is still much room for medicinal chemists to develop the ideal biological tool compounds and drugs for the large variety of P2 receptor subtypes.

## Data Availability

All data are available upon reasonable request.
